# Use of Mediterranean By-Products to Produce Entire Male Large White Pig: Meat and Fat Quality

**DOI:** 10.3390/ani11113128

**Published:** 2021-11-01

**Authors:** Macarena Egea, Irene Peñaranda, María Dolores Garrido, María Belén Linares, Cristian Jesus Sánchez, Josefa Madrid, Juan Orengo, Fuensanta Hernández, María Arantzazu Aguinaga Casañas, Alberto Baños, Belén Barrero Domínguez, Silvia López Feria, Silvia Martínez Miró

**Affiliations:** 1 Department of Food Science and Technology, Veterinary Faculty, University of Murcia, 30071 Murcia, Spain; irene.penaranda@um.es (I.P.); mgarrido@um.es (M.D.G.); blinares@um.es (M.B.L.); 2 Department of Animal Production, Faculty of Veterinary, Campus de Espinardo, University of Murcia, 30100 Murcia, Spain; cristianjesus.sanchez@um.es (C.J.S.); alimen@um.es (J.M.); jorengo@um.es (J.O.); nutri@um.es (F.H.); silviamm@um.es (S.M.M.); 3 DMC Research Center, Camino de Jayena, S/N, 18620 Alhendín, Granada, Spain; arancha.aguinaga@dmcrc.com (M.A.A.C.); abarjona@dmcrc.com (A.B.); 4 Dcoop Sociedad Cooperativa Andaluza, Carretera Córdoba S/N, 29200 Antequera, Málaga, Spain; belen.barrero@dcoop.es (B.B.D.); silvia.lopez@dcoop.es (S.L.F.)

**Keywords:** *Allium* spp. extract, olive pulp, pig feeding, fatty acid profile, antioxidant capacity, sensory analysis, pork

## Abstract

**Simple Summary:**

Sustainability is a current issue that is gaining an important place in consumer choices. Natural and environment-friendly products are becoming more popular and widely accepted, so changes to production methods are needed. Local by-products offer a good way to close the circle on animal production. In this study, two by-products were used: Garlicon ST^®^, a supplement obtained from garlic and onion, which could improve the microbiota gut and the ingestion in pigs, and olive pulp from Mediterranean oil production, which could be a good source of nutrients for pigs. The impact of the use of both by-products on the quality of meat and fat of pigs was examined.

**Abstract:**

A total of 70 male growing non-castrated pigs (Large White), with a 23.07 ± 2.87 kg average body weight (BW), were randomly allocated to three treatments in a 103 day trial: a CONTROL diet and two experimental diets, ALLIUM (5 g/kg of *Allium* spp. extract) and OLIVE (100 g/kg of olive pulp). Animals were slaughtered at 115 kg live body weight. Meat and fat quality were analyzed. Animals fed ALLIUM and OLIVE had higher water holding capacity (WHC) than those fed the control diet. No significant differences were observed between groups for cooking loss, drip losses and color CIELab. No antioxidant effect was observed on an oxygen radical absorbance capacity (ORAC) test. Animals fed OLIVE presented a more unsaturated fatty acid profile than CONTROL and ALLIUM. Meat from ALLIUM group and OLIVE showed her values of brightness and meat odor than CONTROL. Mean scores of sensory analyses (color, odor, flavor and juiciness) of cooked samples were similar for the three treatments, with the meat samples from the ALLIUM and OLIVE treatments being less hard. Consumers did not reflect a preference for any of the treatments. Both by-products could be used for pork production.

## 1. Introduction

The growing awareness of climate change and the impact of our habits on environment have led on an increment of sustainability issues onpublic interest in recent years. Thus, the impact of the food in some aspects as the environmental, ethical, and animal welfare are increasing their presence in the conscientious of consumers them [1]. In this sense, consumer demand for safe and natural animal foods with added nutritional value is a challenge for animal nutrition. There is a trend toward using phytogenic feed with functional properties of natural origin versus potentially harmful synthetics. For this reason, a great deal of interest has been expressed for aromatic plants and other vegetable products, since they are considered an untapped reservoir of valuable substances and their research is an ongoing discipline [2].

Resource consumption to develop livestock production is costly, so substitution of feed grains with other ingredients derived from food waste would help reduce “livestock’s long shadow”. By using current technologies to utilize discarded feed waste and recovered it, the farmland needed for European pig production could be reduced by 20% [3].

In this sense, the use of by-products in animal production is a historical practice, and it has been gaining strength in recent years. Olive by-products are rich in oil and are a good option to improve fat, by reducing medium chain fatty acids and increasing polyunsaturated fatty acids, as well as reducing costs, as they are cheaper than cereals [4]. The main by-products used in animal feed are: olive leaves and olive cake made up of pulp, skin, stones and water. When the stones are removed by mechanical extraction the by-products obtained are olive pulp and olive pomace. The latter is one of the main by-products obtained from olives, since for every 100 kg of olives, 40 kg of pomace are obtained [5]. The olive cake has a high content of crude fiber, oil (unsaturated fatty acids) and antioxidants. The fiber has a limited microbial digestibility due to the high lignin concentration, and thus biodigestion will be low [6]. In addition, olive by-products contain antioxidants, such as tocopherols and retinol and bioactive phenols, which could be interesting for extend shelf life of unsaturated fat, and to improve the nutritional profile of meat [7]. Research on strategies such as dietary supplementation of unsaturated vegetable oils in diets has emerged from the growing consumer demand for safe and healthy foods. In this context, animal feed supplemented with co-products from the agricultural industry could be an interesting strategy both to minimize its ecological footprint as well as to influence the quality of products of animal origin. This case is remarkable in the case of co-products that contain appreciable amounts of vegetable oils, which can contribute to increase the content of unsaturated fatty acids in meat. [7]. Previous studies found that inclusion up to 10% of olive cake on pigs, obtained no negative effect on parameters such as pH or color, although found a reduction of intramuscular fat content. An increaseunsaturated fatty acid profile was observed when animals were fed with olive by-product. There was no too much in relation with sensory impact on meat or consumer aceptance [4].

On the other hand, the harvest sector, having helped shape consumer expectations of “perfect” fruits and vegetables, is now driven to meet the demand, resulting in a huge amount of waste [8]. Garlicon ST^®^ is a sustainable product obtained from garlic and onions rejected by consumers. The use of phytogenic feed additives, such as garlic and onion, has gained increasing attention, due to its antioxidant action, they enhance atability of the diet by increasing the consumption of feed in animals and their growth, in addition to improving intestinal functions and antimicrobial actions. [9]. Over the last two decades, plant extracts use has grown, including in pig feeding, and has been shown to improve carcass and meat quality. Some changes could be observed, as color, althougt it depends on extract composition [10]. It was observed that these changes could be not perceived in sensory analyses, where parameters such as overall color, appearance, taste, odor, tenderness, juiciness, fat sensation and connective tissue were not pointed in prevoius studies [10] However, the use of new ingredients, such as by-products, in pig feeding can lead to anomalous meat flavors, and additional studies are therefore needed [11].

Thus, the aim of this work was to study the effect of the addition of two Mediterranean by-products (*Allium* spp. extract and olive pulp) in pig feeding on the meat and pork fat quality.

## 2. Materials and Methods

### 2.1. Ethical Statement

This research was carried out at the Veterinary Teaching Farm facilities of the University of Murcia, Spain, with the approval of the Animals Experimentation Ethics Committee of the University of Murcia and the Authorities of the Region of Murcia (4 August 2017, n° A-13170805), following the Directive 2010/63/EU of the EU Parliament and of the Council of 22 September 2010 on the protection of animals used for scientific purposes [12].

### 2.2. Supplements and Diet Ingredients

The *Allium* spp. extract (Garlicon ST^®^) used was supplied by DOMCA Co., Granada, Spain. The composition of this organoleptic feed additive is a mix of flavouring substances (7.956 mg/kg) containing garlic and onion extract standardized in propyl propane thiosulfonate (PTSO), an organosulfur compound characteristic of *Allium* species, including sepiolite (E-562) and glyceryl polyethyleneglycol ricinoleate (E-484) as technological additives. GARLICON ST^®^ was added to the animal feed at 5 g/kg (powder form, equivalent to 30 ppm of the active ingredient).

Olive pulp was provided by Dcoop Sociedad Cooperativa Andaluza, Antequera, Spain. Olive pulp was obtained as a by-product from olive oil, and comprised the deboned and dehydrated residue from the oil mill, thus it was composed by remains of pulp and skin of the olive’s fruit. This by-product has a high oil content; its chemical composition was: 8.2, 12.6, 32.9 and 12.1% for crude protein, ether extract, neutral detergent fiber and acid detergent lignin (as dry matter basis), respectively.

### 2.3. Experimental Design, Animals, and Diets

A total of 70 male growing pigs (Large White), with 23.07 ± 2.87 kg of average body weight (BW) were randomly allocated to three treatments in a 103 day trial. Three dietary treatments for each feeding phase were followed: the CONTROL diet and two experimental diets: ALLIUM (5 g/kg of GARLICON ST^®^ to replace sepiolite) and OLIVE (100 g/kg of olive pulp). The diets were based on cereals and soybean meal (Table 1). The olive pulp was incorporated in substitution of cereals, adjusting the energy with lard and more energetic cereals, and the protein with soybean, so that the feeds were designed to be isoenergetic (2.45 and 2.40 Mcal net energy/kg for the growing and finishing phase, respectively) and isoprotein (0.94 and 0.80 g standardized intestinal digestible Lys/kg for the growing and finishing phase, respectively). All the diets were formulated according to the Spanish Foundation for the Development of Animal Nutrition [13]. Throughout the study, the pigs had feed and water ad libitum, and they were managed in commercial conditions.

### 2.4. Slaughter and Sample Collections

Pigs were sacrificed in a commercial slaughterhouse when pigs reached slaughter weight (mean 115 kg live body weight), being previously stunned with CO_2_. After slaughter, the *Longissimus thoracis et lumborum* muscle was extracted from the carcasses and they were packed, identified and transported to the Food Technology Laboratory of the University of Murcia, Spain. The meat was storage at 4 °C during 24 h. The loins had the fat removed and were cut into 1.5 cm fillets. Some of the samples were processed raw for the determination of humidity, fat, WHC and the fresh sensorial analysis; the others were packaged and frozen (−18 °C) for fatty acids and cooking sensorial analysis.

The pH was measured 24 h post-mortem in the carcass, in the *Longissimus thoracis et lumborum* muscle, at the level of the tenth rib, with a portable pH meter (Crison GLP21, Eutech, Singapore, Republic of Singapore) equipped with a penetrating glass electrode.

Fat (using petroleum ether (40–60 °C) as solvent in a Soxhlet extraction) and protein (Kjeldahl N × 6.25) were quantified according to the AOAC (2006) [14]. The WHC was determined by following the Grau and Hamm [15] method, using 1 kg weight and 10 min of pressure for each sample. Whatman n° 54 paper was used for the determination. The results were expressed as a percentage of the initial sample weight. Drip loss was evaluated by packaging the samples in polystyrene trays (B5-37 AerPack; ALIAGA and Ortiz, SL, Alcantarilla, Spain) covered with permeable film (MICAL^®^ professional, Miquel Alimentació Group SAU, Vilamalla, Spain) and stored at 4 °C [16]. The samples were weighed 0, 72 and 120 h after packaging, and drip loss was expressed as a percentage of the initial weight of the sample. All the determinations were done by duplicate.

To evaluate the cooking loss the meat samples (100 g of fillet weight) were cooked in water (in vacuum packaging bags) at 80 °C at an internal temperature of 72 °C. Reference sample was used with bag opened and the fillet was measured with a T200 portable thermometer, Digitron Instrumentation Ltd., Merd Lane, Hertford, UK, up to 72 °C in the center. Then, the samples were tempered at 21 °C before weighing [17]. The percentage of weight loss was calculated using the following formula. The determination was done by duplicate.

$$Cooking\;loss\;\left(\%\right)=\frac{\left(Initial\;weight-final\;weight\right)\times 100}{initial\;weight}$$

The color was expresed by CIELab scale, where a*—redness, b*—yelloness and L* lightness of the meat and they were measured with a Chroma Meter CR-400 (Minolta Ltd., Milton Keynes, UK) calibrated against a standard white tile (8 mm diameter aperture, d/0 illumination system, illuminant D65 and a standard observation angle of 2°). The determination was done by triplicate.

For the oxygen radical absorbance capacity (ORAC) analysis the sample preparation was carried out according to the method described by Huang et al. [18] and the preparation of the plate and the ORAC test were carried out following the protocol of Cao and Prior [19]. Results were expressed as micromol Trolox equivalents per kilogram (wet). All the determinations were done by duplicate.

### 2.5. Fatty Acid Profile Analysis

Fatty acids were analyzed in feed and meat using the UNE-EN ISO 5508 method and by gas chromatography [20]. The fat was extracted using a cold decantation method [21] using Folch’s solution (chloroform:methanol) 2:1 and butylhydroxytoluene (BHT). For methylations, 0.2 N sodium methylate was added and stabilized by adding a 3% solution of sulfuric acid in anhydrous methanol. Finally, n-hexane was added and reheated to promote dissolution of the esters. Subsequently, the injection was carried out in the gas chromatograph (Thermo Finnogan Trace GC Ultran, Milan, Italy) using an autosampler (Thermo Scientific AS 3000, Milan, Italy) and a polar TR-CN100 capillary column (Teknokroma, Barcelona, Spain). For the separation of fatty acids, three temperature ramps were used, from 70 to 250 °C, and helium was used as a carrier gas (flow of 3.2 mL min^−1^). The methyl esters were identified by the retention times of the reference standards (FAME mix 37 from Sigma Aldrich, Darmstadt, Germany) and were quantified using the methyl ester of undecanoic acid as an internal standard and with the calibration lines of each fatty acid. Atherogenic index and thrombogenic index were calculated following Chen and Liu [22]. All the determinations were done by duplicate.

### 2.6. Sensory Analysis

Three sensory analyses were carried out: Two of them with a trained panel, where fresh and cooked samples were analyzed by quantitative descriptive analysis, and the other one with a consumer panel to evaluate preference. For the first two, panel selection and training were made according to ISO 8586-2 [23]. Eight trained panelists from the Murcia University were selected. Six theoretical-practical sessions of 1.5 h were held for specific training on each of the products (fresh meat and cooked meat). The first session was carried out with the raw meat samples, 48 h after slaughter, where the following parameters were evaluated: color intensity, brightness, marbling and odor intensity (Table 2). The samples were kept at room temperature (23 °C) for 10 min to oxygenate. For the cooked trial, the samples were cooked at 150 °C on a double plate griddle (Silanos, Liscia Average, Lavastoviglie Industriali, Italy) until the center of the product reached 72 °C (T200 portable thermometer, Digitron Instrumentation Ltd., Merd Lane, Hertford, UK). The fat was removed from the edges and cut into squares (2 cm × 2 cm), which were wrapped in aluminum foil and kept warm in a sand bath (Braun, Esplugues de Llobregat, Spain). The samples were supplied to the trained panel at random, without exceeding six samples per day. Each panelist analyzed a total of three samples per treatment. For both sessions, the fillets were cut with a thickness of approximately 1.5 cm. Descriptive analysis was carried out using a 10 cm unstructured scale for fresh and cooked meat. All sessions were made following the ISO 4121 [24] standard, in a standardized room of the Department of Food Technology of the University of Murcia that met the conditions established in the UNE EN-ISO 8589 [25]. Mineral water (La Serrata, Valencia, Spain) and unsalted bread (Aliada, Madrid, Spain) were provided for mouth rinsing between samples. The following parameters were evaluated: color intensity, odor intensity, off odor, flavor intensity, off flavor, hardness, juiciness and chewiness (Table 2).

For consumer tests, the samples were prepared in the same way as that for the cooked sensory analysis. A total of 60 consumers were recruited randomly among students, professors and workers from the University of Murcia (Campus de Espinardo, Spain). The consumers ranked the samples according to their own preference [26]. Then, they had to indicate if their answer was based on color, odor, hardness, juiciness and/or flavor of the meat sample.

### 2.7. Statistical Analyses

A one-way ANOVA analysis was performed, with dietary treatment (Control, ALI and OLIVE) as a fixed effect, using SPSS version 24.0 software (SPSS Inc., Chicago, IL, USA). Tukey’s test for post-hoc was applied. For the consumer sensorial test, the Friedman rank sum test was performed, using a significance level of 95% to determine whether the panelists were able to discriminate among samples. Data were assigned with value in relation with a decreasing preference order, 1 for first, 2 for second and 3 for third. All the values were added and included in the formula below. The least significant difference was used to determine whether significant differences (*p* ≤ 0.05) existed among treatments.

## 3. Results

### 3.1. Meat Quality

Table 3 shows the effects of the addition of the by-products tested to pig diets on meat quality parameters. Low levels of intramuscular fat were found in meat in this trial (1.02% for the CONTROL, 1.04% for the ALLIUM, and 1.18% for the OLIVE treatment), and probably due to the use of males of the Large White breed. Large White is a maternal line, characterized by its prolificacy and better maternal characteristics, but by contrast, these animals produce leaner meat. Sellier et al. [27] analyzed the intramuscular fat content of more than 1000 samples of Longissimus dorsis from white pigs (Large White and Landrace) and obtained a mean value of 1.23 ± 0.46 g/100 g of muscle. These facts, combined with no castration of male pigs, could be responsible of the low fat content. Carcasses from entire males (non-castrated) are leaner than those from castrated males and females, and they showed that the ratio of intermuscular to subcutaneous fat for entire males is higher than in castrates [28].

Meat from treatment groups showed a higher WHC (70.22 and 71.89%, for ALLIUM and OLIVE, respectively) than the control group (67.98%). On the contrary, García-Casco et al. [29] found that the control batch presented more WHC than the batch from pigs fed with wet alperujo, another olive oil by-product. These differences may be due to the type of by-product used. Other studies found no differences, such as Joven et al. [30], between the control batch and batches of pigs fed with increased levels of olive cake. Park et al. [31] studied different fat sources for pig feeding and also found no significant differences (*p* > 0.05) between groups fed olive oil, coconut oil, soybean oil or beef tallow.

No significant differences were observed between groups for the rest of the parameters studied: cooking loss, drip losses, color CIELab and ORAC. The ORAC value was expressed as micro moles of Trolox Equivalents (TE) per kilogram of sample, an analog of vitamin E that, due to its easy solubility in water, is used as a standard of comparison. Although the administered by-products come from plant materials rich in polyphenolic compounds with known antioxidant activity, its administration endogenously was not enough to see any effect on meat oxidation. In addition, fat that could store these compounds was very low, and thus better results would be expected in fat breads such as Iberian pigs. Janz et al. [32] found no significant differences with respect to oxidative stability of meat in pigs fed essential oils and oleoresins from different herbs, including garlic.

### 3.2. Fatty Acid Profile

Table 4 presents results obtained from the fatty acid profile of the meat of pigs fed the three types of diets. Differences (*p* ≤ 0.05) were observed in the percentage of C17:0, C18:0 and C18:3. Margaric acid (C17:0) had a higher value (1.08%) for the batch of animals fed ALLIUM than animals fed with OLIVE. By contrast, linolenic acid was lower in the ALLIUM than the CONTROL and OLIVE groups. This type of fatty acids with an odd number of carbon atoms is typical in ruminant fat and is derived from volatile fatty acids (VFAs) absorbed from microbial fermentations in the rumen. In monogastric animals, VFAs are produced by microbial fermentations in the large intestine; therefore, these differences between treatments highlight the effects of different diets on the microbiota of the large intestine of pigs. Regarding linolenic acid, we did not find an explanation for this effect, although the proportion and magnitude of the decrease was very low. The incorporation of olive pulp led to the lowest levels of stearic acid (*p* < 0.05), while a tendency was observed (*p* = 0.076) to raise the percentage of oleic acid. Pigs fed olive pulp showed a more unsaturated fat profile (*p* ≤ 0.05) than the other groups. Our results agree with those reported by Joven et al. [30], who observed in subcutaneous fat of pigs fed with increasing amounts of olive cake (incorporated in the range of 0–15% into feed) a reduction in margaric acid (C17:0) and stearic acid (C18:0), and an increase in oleic acid; specifically, oleic acid ranged from 41.2% in the fat of control pigs to 41.7% in pigs with 10% of olive pulp in their feed, an lower improvement than that observed in our study (41.9 vs. 43.7% for the CONTROL and OLIVE groups, respectively).

On the other hand, the reduction in the content of saturated fatty acids in the fat of pigs fed with olive pulp coincides with that observed by Doyle et al. [33] in pigs fed with olive pomace during the finishing phase. Likewise, Hernández-Matamoros et al. [34], when feeding Iberian pigs with alperujo (55% in the ration for 28 days), also found a reduction in saturated fatty acids in the subcutaneous fat of pigs, and an increase in the oleic acid level.

There were no differences between groups for index of atherogenic. A low atherogenic value is desirable since indicates a lower proportion of saturated acids, and reduces plaque formation on blood vessels. Both werein the usual range for meat (0.165 to 1.32 for index of atherogenic, and 0.288–1.694 for index of thrombogenic) [22]. Index of thrombogenic showed significant differences in the OLIVE group respect CONTROL and ALLIUM groups. This index indicates a lower risk to blood coagulation process incidents [22]. The ratio unsaturated:saturated fatty acid was higher in the OLIVE group than in CONTROL and ALLIUM. The ratio of unsaturated fatty acids to saturated in the diet should be 1:1. according to FAO 2010 recommendations. CONTROL and ALLIUM groups obtained 1.5 points, while OLIVE group obtained 1.6 [35].

### 3.3. Sensory Analysis

Table 5 shows the results of the sensory analysis of fresh meat corresponding to the treatments tested, scored according to an unstructured scale of 10 cm. No significant differences were found for color intensity and marbling. The results in meat color differed from those obtained by García-Casco et al. [29], since the animals fed with dry alperujo had paler meats and a lower myoglobin content; however, the same research also found no variation in the marbling parameter among the animals fed wet or dry alperujo.

**Figure 1:**
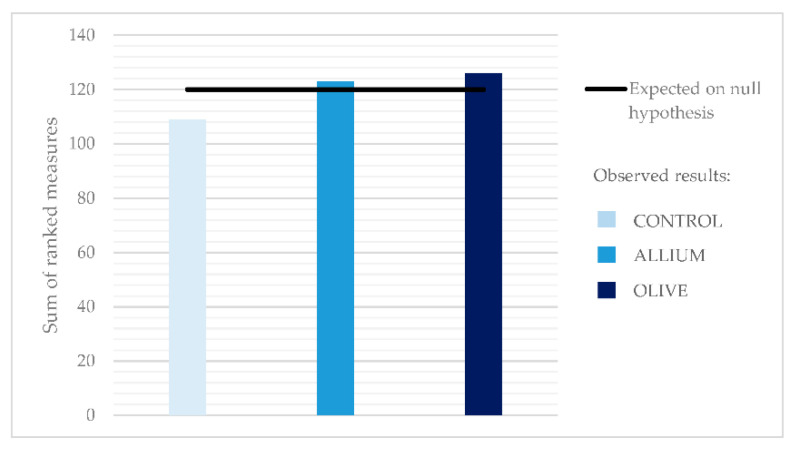
Rank-ordered results of consumer preference (by decreasing order from 1 to 3) for the treatments obtained in the ranking test (*n* = 60). Results are expressed as the sum of rank scores of consumers (*p* > 0.05 based on Friedmans test).

**Figure 2:**
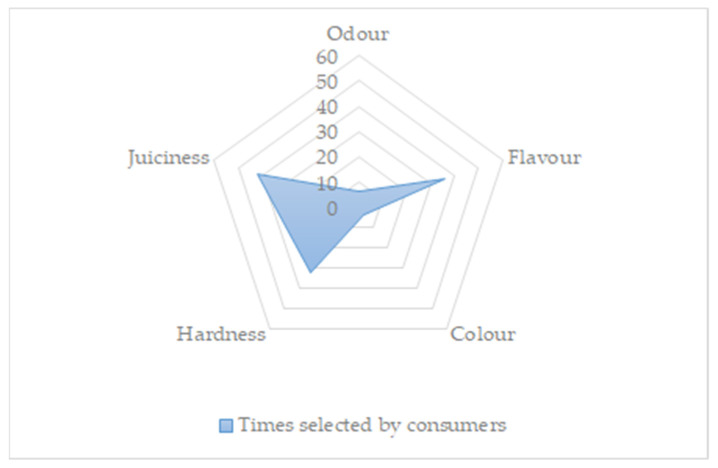
Radar chart with the results about principal parameters influencing the decision of consumers (*n* = 60) on the ranking test. Results are expressed as the sum of consumers selecting each parameter.

Significant differences were found between batches for brightness and odor intensity (*p* < 0.01). Although the ANOVA results showed significant differences, the mean value of each did not vary in most cases by more than one point, and these attributes had less influence on consumers overall quality than others, such as juiciness or hardness (see Figure 1 and Figure 2). On the other hand, in both the ALLIUM and OLIVE diets, the brightness was higher than in the control. By contrast, other studies found that inclusion of olive by-products reduced the brightness of meat [30]. The higher brightness of the olive batch may be due to the higher content of unsaturated fatty acids (Table 3).

As a positive property, a higher odor was perceived in both by-product-based diet groups, which could be explained by metabolites that could be absorbed in guts and, then, pass on to muscles, constituting a matrix for precursors of several volatile compounds responsible for the characteristics of meat odor. Despite raw meat being characterized by a very weak odor, it contains free sulfur amino acids, which results in the formation of many key volatile sulfur compounds, such as bis(2-methyl-3-furyl)disulfide, 2-furanmethanothiol, 2-acetyl-2-thiazoline and 2-methyl-3-furanthiol, with characteristics meaty flavor notes [36,37]. In this sense, the organosulfur compounds present in alliaceae extract can take part in the reactions of synthesis of these molecules by means of their thiol groups, which can explain the higher odor intensity in the ALLIUM group. A similar process could occur with olive volatile compounds, aldehydes, alcohols, esters, hydrocarbons, ketones, and furans which could be perceived by panelist [38].

The low marbling values found are related to the low fat content previously mentioned, since the carcasses of whole males have greater muscle development and a lesser thickness of subcutaneous fat. This is due to androgens that are produced at the testicular level that produce an increase in protein synthesis and consequently a decrease in fat. Specifically, entire (non-castrated) males had 5% less extractable fat and 1% more protein than castrated males [39].

The results from cooked sensory analyses assessed by a trained panel are presented in Table 6. No significant differences were observed for any of the sensory attributes analyzed in the cooked meat, except for hardness. Although other authors have reported changes in the odor and flavor of meat [40], our results do not show these changes when using this standardized alliaceae extract in cooked meat. The treatments with alliaceae extract and olive pulp had the lowest hardness values. In general, the results showed pale meats, with high toughness and low juiciness. The tenderness of the meat depends on its ability to retain water [41], and thus toughness and tenderness are inversely related. It can be considered that the decrease in toughness in the batch of pigs fed with ALLIUM and OLIVE may be due to their greater water retention capacity (Table 2).

Nuernberg et al. [42] determined that supplementation with 5% olive oil did not affect the juiciness and the general flavor of the pork, which is in agreement with the current findings (Table 6). They also found no differences in the meat tenderness, and therefore the toughness was not affected by the diet supplied.

Figure 1 shows the results obtained from the consumer test. The study was carried out by 60 consumers, of which 51.66% were women and 48.33% men. All of them were aged more than 18 years, and segmented for the study into two groups: 18–40 (53.34%) and >40 years (46.66%). There were no differences (*p* > 0.05) for the samples analyzed by the consumers. The ranking selected was mainly focused on juiciness, hardness and flavor (Figure 2). Felderhoff et al. [43] found that flavor was the largest contributor to satisfaction in comparison with tenderness or juiciness in beef.

Although some authors have reported that the incorporation of dietary garlic into the feed transmits its characteristic flavor to meat [44,45], as some flavor components of the diet can be stored in the water or cytoplasm portion of the fat adipose cells influencing the overall taste [46], our results show that there was no transfer of the characteristic flavor of allium to the meat when using Garlicon ST^®^. These discrepancies can be explained by the fact that the previous authors used whole dried garlic cloves that contain many different compounds from allicin degradation whose composition in active compounds totally differs from the allium extracts used in our trials, whose active ingredient are well characterized and are rich in propyl propane thiosulfonate. Indeed, Panea and Ripoll [47] reported higher taste scores in pork meat from animals fed with allium extract rich in propyl propane thiosulfonate in consumer preference panels. In addition, allium extracts rich in propyl propane thiosulfonate have been demonstrated not to alter organoleptic properties in eggs or milk when added to the diet of animals [48,49].

## 4. Conclusions

The olive pulp and *Alium* spp. extract used in pig feeding had no negative effects on physical, chemical or sensorial meat parameters. In addition, both by-products improved the WHC of the meat, which could be positive for consumer that usually reject meats with high drip loss. The use of olive pulp increased the unsaturated fatty acid profile, which could be interesting for consumers’ health concerns. At the sensorial level, neither Alliaceae extract nor olive by-products gave off a strange odor or flavor. In addition, the by-product diets seemed to reduce meat hardness entire male animals. Finally, consumers seemed to present the same preference to that of the control. In conclusion, both sustainable options (Garlicon ST^®^ and olive pulp up to 10%), could be included in the diets of white pigs and their use in the feeding of fatter pig breeds could be of interest.

## Figures and Tables

**Table 1:** Diet composition.

Ingredient (% of Feed)	CONTROL		ALLIUM		OLIVE	
	Growing	Finishing	Growing	Finishing	Growing	Finishing
Barley	74.57	10.74	74.57	10.74	-	2.39
Corn	-	41.19	-	41.19	40.00	47.43
Wheat	-	15.00	-	15.00	21.99	15.00
Soybean meal	17.72	15.55	17.72	15.55	20.68	17.76
Wheat bran	-	10.00	-	10.00	-	10.00
Olive pulp	-		-		10.00	10.00
Lard	3.67	4.03	3.67	4.03	3.48	4.12
Calcium carbonate	0.82	1.11	0.82	1.11	0.54	0.79
Monocalcium phosphate	0.34	0.78	0.34	0.78	0.45	0.99
Salt	0.50	0.41	0.50	0.41	0.50	0.40
DL-Methionine	0.10	0.05	0.10	0.05	0.10	0.05
L-Lysine HCl	0.33	0.28	0.33	0.28	0.31	0.21
L-Threonine	0.12	0.06	0.12	0.06	0.11	0.05
Dextrose	1		1		1	
Garlicon ST^® 1^	-	-	0.5	0.5	-	-
Sepiolite	0.50	0.50	-	-	0.50	0.50
Premix ^2^	0.30	0.30	0.30	0.30	0.30	0.30
Fatty acid profile in fat (%)						
Capric acid [C10]	0.05	0.06	0.06	0.07	0.05	0.07
Lauric acid [C12]	0.09	0.07	0.09	0.10	0.07	0.08
Myristic acid [C14]	0.95	0.83	1.01	0.76	0.67	0.79
Palmitic acid [C16]	22.95	21.60	23.39	20.88	21.04	20.92
Palmitoleic acid [C16:1]	1.47	1.42	1.53	1.35	1.20	1.48
Stearic acid [C18]	9.56	8.86	9.47	8.69	8.94	8.75
Oleic acid [C18:1]	34.71	35.64	34.28	38.42	40.79	42.20
Linoleic acid [C18:2]	26.94	28.97	26.88	27.31	24.92	23.65
Linolenic acid [C18:3]	2.41	1.78	2.43	1.66	1.60	1.38
Arachidic acid [C20]	0.70	0.60	0.66	0.58	0.55	0.52
Arachidonic acid [C20:4]	0.18	0.18	0.20	0.19	0.17	0.15

^1^ *Allium* spp. extract, rich in propyl propane thiosulfonate (PTSO), is marketed under the trademark Garlicon^®^ and was added to the feed in powder form. ^2^ The premix provided the following vitamins and minerals (per g of diet): vitamin A, 6000 IU; vitamin D3, 450 IU; vitamin E, 15 IU; vitamin K3, 0.80 mg; biotin, 0.01 mg; vitamin B1, 1.0 mg; vitamin B2, 2.5 mg; vitamin B6, 1.5 mg; vitamin B12, 0.015 mg; nicotinic acid, 15 mg; pantothenic acid, 10 mg; choline chloride, 100 mg; Mn, 25 mg as MnO; Zinc, 90 mg as ZnO; I, 0.40 mg as KIO_3_; Cu, 20 mg as a cupric chelate of glycine hydrate; Se, 0.20 mg as Na_2_SeO_3_; Fe, 60 mg as FeCO_3_; 6-phytasa, 0.40 FTU; xylanase, 0.56 TXU; beta-glucanase, 250 TGU.

**Table 2:** Attributes used in sensory analysis.

Attribute	Description	Scale
Color Intensity	Intensity of red color in fresh meat and grey in cooked meat.	0 pink–10 red 0 white–10 grey
Brightness	Reflection of light on the surface of the product.	0 light–10 dark
Marbling	Infiltrated visible fat in loin.	0 not fat–10 high fat content
Odor intensity	Characteristic odor of pork (metallic, farm, liver).	0 low intensity–10 high intensity
Off odor	Any odor that cannot be included in “meat odor”.	0 not present–10 strong presence
Flavor intensity	Intensity of the perception of the characteristic flavor of pork.	0 low intensity–10 high intensity
Off flavor	Any flavor that cannot be included in “meat flavor”.	0 not present–10 strong presence
Juiciness	Parameter that measures the amount of water released by the product in the first bites.	0 not juicy–5 juiciness of commercial product–10 high juicy
Hardness	Force necessary to deform the product between the molars at the first bite.	0 tender–5 commercial sample–10 hard
Chewiness	Number of chews required to swallow a product.	0 less chews–5 number of chews needed for commercial product–10 more chews

**Table 3:** Mean scores of the meat quality parameters analyzed in loin from pigs fed three different treatments.

	CONTROL	ALLIUM	OLIVE	SEM	*p*-Value
Intramuscular fat (% *w*/*w*)	1.02	1.04	1.18	0.057	0.496
pH	5.58	5.60	5.66	0.025	0.403
WHC (%)	67.98 ^a^	70.22 ^b^	71.89 ^b^	0.350	0.000
Cooking loss (%)	33.88	32.97	32.67	0.292	0.252
Drip loss (%)	3.37	3.37	3.46	0.144	0.841
L*	54.47	54.24	54.16	0.208	0.885
a*	5.49	5.34	5.59	0.121	0.634
b*	2.95	2.86	2.66	0.069	0.309
ORAC (micro mols TE/Kg)	61.24	50.83	55.42	2.211	0.236

^a,b^ The means with different letters are significantly different at *p* ≤ 0.05 by Tukey’s test. WHC: water holding capacity. w: weight. Sample size (*n*): 70 pigs (23–24 animals per treatment).

**Table 4:** Fatty acid composition (%) of intramuscular fat from *Longissimus thoracis et lumborum* muscles of pigs fed three different treatments.

		CONTROL	ALLIUM	OLIVE	SEM	*p*-Value
Capric acid	[C10:0]	0.11	0.11	0.12	0.004	0.616
Lauric acid	[C12:0]	0.11	0.11	0.13	0.006	0.094
Myristic acid	[C14:0]	1.20	1.34	1.24	0.032	0.229
Palmitic acid	[C16:0]	25.0	24.8	24.6	0.105	0.186
Palmitoleic acid	[C16:1]	2.61	2.83	2.75	0.054	0.272
Margaric acid	[C17:0]	0.70 ^ab^	1.08 ^a^	0.41 ^b^	0.056	0.000
Stearic acid	[C18:0]	12.2 ^a^	12.0 ^a^	11.4 ^b^	0.089	0.002
Oleic acid	[C18:1]	41.9	42.2	43.7	0.324	0.076
Linoleic acid	[C18:2]	12.6	12.2	12.1	0.344	0.818
Linolenic	[C18:3]	0.72 ^a^	0.54 ^b^	0.73 ^a^	0.017	0.000
Arachidic acid	[C20:0]	0.31	0.32	0.30	0.010	0.693
Arachidonic acid	[C20:4]	2.18	2.28	2.30	0.127	0.932
Saturated (SFA)		39.8 ^a^	39.8 ^a^	38.2 ^b^	0.160	0.000
Monounsaturated		44.5	45.1	46.4	0.364	0.108
Polyunsaturated		15.5	15.0	15.2	0.409	0.858
Unsaturated (UFA)		60.1 ^b^	60.1 ^b^	61.7 ^a^	0.160	0.000
Index of atherogenic		0.56	0.56	0.53	0.007	0.331
Index of thrombogenic		1.2 ^a^	1.2 ^a^	1.1 ^b^	0.009	0.001
UFA/SFA		1.5 ^a^	1.5 ^a^	1.6 ^b^	0.012	0.000

^a,b^ The means with different letters are significantly different at *p ≤* 0.05 by Tukey’s test. Sample size (*n*): 70 pigs (23–24 animals per treatment).

**Table 5:** Means of the sensory attributes analyzed in fresh pork loin of pigs fed with different treatments (0–10 cm scale).

	CONTROL	ALLIUM	OLIVE	SEM	*p*-Value
Color intensity	7.06	7.46	7.57	0.120	0.198
Brightness	8.62 ^a^	9.27 ^b^	9.45 ^b^	0.086	0.000
Marbling	0.74	0.64	1.03	0.083	0.144
Odor intensity	8.77 ^a^	9.85 ^c^	9.21 ^b^	0.089	0.000

^a–c^ The means with different letters are significantly different at *p* ≤ 0.05 according to Tukey’s test. Sample size (*n*): 24 data per treatment (8 panelists with 3 samples each).

**Table 6:** Mean scores of the sensory attributes analyzed in cooked pork loin fed with three different treatments (0–10 cm scale).

	CONTROL	ALLIUM	OLIVE	SEM	*p*-Value
Meat color	9.74	9.88	9.74	0.051	0.076
Color intensity	4.85	5.01	5.00	0.092	0.184
Odor intensity	8.49	8.84	8.50	0.085	0.215
Off odor	0.76	0.84	0.64	0.092	0.542
Flavor intensity	8.13	8.43	7.99	0.111	0.430
Off flavor	0.74	1.02	0.60	0.117	0.207
Hardness	7.18 ^a^	6.29 ^b^	6.73 ^b^	0.121	0.039
Juiciness	4.17	4.44	4.42	0.173	0.120
Chewiness	6.84	6.27	6.78	0.112	0.592

^a,b^ The means with different letters are significantly different at *p* ≤ 0.05 according to Tukey’s test. Sample size (*n*): 24 data per treatment (8 panelists with 3 samples each).
